# 

**Published:** 1847-08

**Authors:** 


### WESTERN JOURNAL

OF

### MEDICINE AND SURGERY.

### EDITORS.

DANIEL DRAKE, M. D.,

PROFESSOR OF PATHOLOGY AND THE PRACTICE OF MEDICINE IN THE UNIVERSITY OF LOUISVILLE.

LUNSFORD P. YANDELL, M. D.,

PROFESSOR OF CHEMISTRY AND PHARMACY IN THE SAME.

THOMAS W. COLESCOTT, M. D.

POSTAGE ON THIS JOURNAL.

Our attention has been called to an error committed by the printer on the cover of two back numbers of this Journal respecting its postage. The error is corrected in the present number. The postage on such periodicals for the first ounce is two-and-a-half cents, and one cent for each additional ounce.

RECEIPTS OF MEDICAL JOURNAL FOR JULY, 1847.

| | |
|---|---|
| Dr. W. Kinney, Millersburg, Ky. | $5 00 |
| —— Dodd, Bolivar, Miss. | 2 00 |
| C. V. Jones, Covington, Ind, | 20 00 |
| J. J. Daniel, Pleasant Grove Tenn., | 10 00 |
| J. S. Baxter, Perryville, Ind, | 5 00 |
| W. C. Humphreys, Gadsden, Ala., | 5 00 |
| T. Goodall, Tompkinsville, Ky. | 5 00 |
| T. M. Ward, Canton, Miss. | 5 00 |
| S. R. Beck, Albany, N. Y., | 5 00 |
| W. H. Wishard, Greenwood, Ind., | 23 00 |
| ——— Massie, Fanthorp's Tenn. | 5 00 |
| J. M. Wells, Washington Ind., | 5 00 |
| B. F. Richardson, Pine Bluff, Ark. | 5 00 |

TRAVELING AGENTS FOR THE WESTERN JOURNAL OF MEDICINE AND SURGERY.

Jno. F. Johnston and Jas. H. Stevenson, Kentucky—C. W. James, Ohio, Indiana, Illinois, Missouri, and Michigan—Geo. C. Brett, Tennessee—Wm. H. Weld & Son, Mississippi and Louisiana.

The Western Journal of Medicine and Surgery is published monthly by the undersigned, at the corner of Main and Fifth streets, Louisville, at $5 per annum, payable in advance. Each number contains 96 pages, making two volumes in the year of more than 550 pages each.

Contributions to its pages by the Physicians of the Valley of the Mississippi, addressed to the Editors, are respectfully solicited.

Letters on business, to be addressed, postage paid, to the publishers.

*January*, 1844. PRENTICE & WEISSINGER.

